# Illness Perception and Depressive Symptoms among Persons with Type 2 Diabetes Mellitus: An Analytical Cross-Sectional Study in Clinical Settings in Nepal

**DOI:** 10.1155/2015/908374

**Published:** 2015-07-07

**Authors:** Suira Joshi, Raja Ram Dhungana, Usha Kiran Subba

**Affiliations:** ^1^Ministry of Health and Population, Nepal; ^2^Nepal Health Research Council, Kathmandu, Nepal; ^3^Psychology Department, Tribhuvan University, Kathmandu, Nepal

## Abstract

*Background.* This study aimed to assess the relationship between illness perception and depressive symptoms among persons with diabetes. *Method.* This was an analytical cross-sectional study conducted among 379 type 2 diabetic patients from three major clinical settings of Kathmandu, Nepal. *Results.* The prevalence of depressive symptoms was 44.1% (95% CI: 39.1, 49.1). Females (*p* < 0.01), homemakers (*p* < 0.01), 61–70 age group (*p* = 0.01), those without formal education (*p* < 0.01), and people with lower social status (*p* < 0.01) had significantly higher proportion of depressive symptoms than the others. Multivariable analysis identified age (*β* = 0.036, *p* = 0.016), mode of treatment (*β* = 0.9, *p* = 0.047), no formal educational level (*β* = 1.959, *p* = 0.01), emotional representation (*β* = 0.214, *p* < 0.001), identity (*β* = 0.196, *p* < 0.001), illness coherence (*β* = −0.109, *p* = 0.007), and consequences (*β* = 0.093, *p* = 0.049) as significant predictors of depressive symptoms. *Conclusion.* Our study demonstrated a strong relationship between illness perception and depressive symptoms among diabetic patients. Study finding indicated that persons living with diabetes in Nepal need comprehensive diabetes education program for changing poor illness perception, which ultimately helps to prevent development of depressive symptoms.

## 1. Introduction

Diabetes mellitus is now emerging as a global epidemic. The problem is growing so rapidly that it is projected to be the seventh leading cause of death by 2030 [1]. The escalating burden of diabetes has already surfaced in low and middle income countries (LMCs) where four out of five people with diabetes are currently living [2]. Recent population based studies conducted in Nepal have also reported a growing prevalence of diabetes among urban, middle, and elderly population [3–5].

Depression, one of the major comorbid conditions associated with chronic diseases [6, 7], has a bidirectional relationship with diabetes [8, 9]. The presence of depression is two times more common in people with diabetes than in those without diabetes [10, 11]. It adversely affects the diet and treatment adherence, glycemic control, and productivity [12]. Therefore, the coexistence of depression among people with diabetes worsens the disease outcomes [13].

In diabetic patients, depression has been linked with the multiple factors like female sex, younger age, not having a spouse, poor social support, and low socioeconomic status, among others [14, 15]. Most importantly, depression is associated with the perception of patients about their disease [12, 16]. Illness representation or perception reflects the patients' own views about the cause (beliefs about how the disease occurred), illness identity (beliefs about how the disease should look like, by relating to the symptoms), illness consequences (impact of the disease on quality of life, relationships, and work), timeline (whether the disease is of long or short duration or has cyclical onset of symptoms), and cure or control (whether the illness can be controlled by patient's behavior or treatment module) [17]. Positive illness perception is established when the illness is viewed as a normal part of life [16, 18]. Curtailed information about the disease collected from the social interactions, from the authoritative sources such as doctors, from patients, and from his/her own experiences forms an overview regarding the disease [19]. Such image may generate the pessimistic view about disease that can lead to psychological disorders [18, 20]. On the contrary, the appropriate knowledge about the condition may provide healthy judgment regarding behaviors related to the disease [20]. Literatures have shown relationship between illness perception and depression in cardiovascular disease (CVD) and renal diseases [21, 22]. Nepal, however, has dearth of similar studies in diabetic patients. Therefore, this research aimed to identify the relationship between illness perception and depressive symptoms in patient with diabetes.

## 2. Methods

### 2.1. Study Setting

This was an analytical cross-sectional study conducted between December 2013 and June 2014 in clinical settings in Kathmandu, Nepal. Study sites were Tribhuvan University Teaching Hospital, Maharajgunj; Diabetes, Thyroid Care and Endocrinology Center, Kupondole; and Diabetic Society Clinic, Jamal. Sites were selected purposively as they were the well-known diabetic treatment centers in Nepal.

### 2.2. Participants, Sample Size, and Sampling Method

Study participants were patients with the diagnosis of type 2 diabetes mellitus attending study sites during the study period. Sample size was calculated using *Z* ^2^ · *pq*/*d* ^2^ formula [23] at 95% confidence interval and 5% allowable error. The estimated prevalence of depression among persons with diabetes was assumed to be 40.3%, based on previous similar hospital based study [14]. Total sample size came out to be 390.

Samples were proportionately allocated to each study site with respect to the average number of patient visits to the centers in previous months. Participants between 20 and 70 years of age attending study sites with present history of diabetes of at least six months of duration were selected for the study. The total number of eligible patients registered in all study sites was 1200. Among them, every third participant was enrolled in the study.

Patients with diabetic complications like neuropathy, nephropathy, retinopathy, autonomic neuropathy, and so forth were excluded because of their independent association with depression [14]. Likewise, those with the previous episode of depression or using any antidepressant medication were also excluded.

### 2.3. Ethical Approval

Ethical approval was taken from the Nepal Health Research Council (NHRC), Ramshah Path, Kathmandu, Nepal. Each participating institute also reviewed the study protocol and provided consent for conducting research. Purpose of the study was clearly explained to all the respondents. They were well informed about their right to withdraw from the study at any time. Participants were also assured that all personal details would be confidential. Considering no more than minimal risk in research participation, verbal consent was taken prior to an interview. Consent procedure was approved by Ethical Review Board of the NHRC based on National Ethical Guidelines for Health Research in Nepal. No financial incentive was provided to the participants.

### 2.4. Instruments

The first section of the data collection tools was structured questionnaires, designed to collect the sociodemographic data. Age was recorded as the completed age of participants at the day of interview. Occupations were recorded in different categories: job (government or nongovernment), business, farming, and so forth. Kuppuswamy's Scale was used to categorize socioeconomic status of the participants. It utilizes the level of education, household income, and occupation for calculating individual's socioeconomic status [24]. Participants were also requested to report the diabetes duration, use of antidiabetic medications (insulin/pills) including alcohol consumption, and cigarette smoking habit.

### 2.5. Illness Perception Questionnaire-Revised (IPQ-R)

The well validated Illness Perception Questionnaire-Revised (IPQ-R) was used for assessing the illness perception [25]. The IPQ-R was presented in three sections. Firstly, diabetes identity was assessed using 14 commonly experienced symptoms like headache, joint stiffness, weight loss, and so forth. For each symptom, participants were asked to indicate (a) if they had experienced this symptom since their diabetes and (b) whether they believed the symptom was related to their diabetes. The responses were recorded as yes/no. The sum of the yes-rated symptoms on the second scale comprises the diabetes identity subscale. Timeline acute/chronic, timeline cyclical, consequences, personal control, treatment control, illness coherence, and emotional representations were assessed in the second section. Altogether 38 items were presented with a five-point response scale: strongly disagree, disagree, neither agree nor disagree, agree, and strongly agree. Lastly, in the third section, 18 possible causes of diabetes were listed. Responses were recorded on a five-point scale as mentioned above. The scale was scored accordingly: strongly disagree = 1, disagree = 2, neither agree nor disagree = 3, agree = 4, and strongly agree = 5. Identity was scored as yes or no, where yes = 1 and no = 0.

### 2.6. Beck Depression Inventory-II (BDI-II)

BDI-II scale was administered to assess depressive symptoms among the participants. It contained 21 questions, each answer being scored on a scale value of 0 to 3. For clinical interpretation, presence of depressive symptoms was defined as a BDI-II score ≥14 [26, 27]. The tool is not validated for use in Nepal. However, it has been previously used in Nepal for assessing depression level [28]. We opted to use the tool in our study, considering its latest improvisation as per DSM-IV criteria for major depression [29]. BDI-II omits items relating to weight loss, body image, hypochondria, and working difficulty and has added items relating to agitation, worthlessness, difficulty concentrating, and energy loss compared to earlier versions in order to assess the intensity of depression [30, 31].

### 2.7. Translation and Pretesting

The questionnaires were translated into Nepali by two multilingual researchers individually. Translations were compared to identify the discrepancies. Principal investigator mediated the discussion between two translators and facilitated coming up with single version. Another bilingual researcher back-translated it into English and validated it with the original one. Two experts from Psychology Department of Tribhuvan University consolidated the different versions of translations making them equivalent to source version. Effort was made to ensure that the translation carried the same meaning as the original version while also being sensitive to the nuances of Nepali language. The translated version was pretested among 20 respondents.

### 2.8. Reliability of the Instruments

For assessing reliability of both instruments (illness perception, BDI-II), we calculated reliability coefficients (Cronbach's alpha) for all nine subscales of illness perception and BDI-II using the study dataset.

### 2.9. Training

Five research assistants were chosen and trained to administer the questionnaire in seven- day training program. They were asked to repeat the mock interviews under direct supervision of investigators until desired standards were achieved.

### 2.10. Data Management and Analysis

Data were compiled, edited, checked, and validated to maintain consistency. Collected data were entered in Epidata V.2.1 and exported to SPSS V.16.0 for further analysis.

Distribution of sociodemographic characteristics by gender was presented in table with frequency and percentage. Chi-square test was conducted to compare the proportions of depressive and nondepressive symptoms among respondents across the sociodemographic strata. Spearman's correlation was applied for analyzing the relationship between illness perception and depression scores. All tests were two-tailed and *p* < 0.05 was considered statistically significant.

Multivariable analysis was conducted using binary logistic regression. Presence of depressive symptoms was considered as dependent variable with dichotomous outcomes: 0 = absent, 1 = present. Those variables that were significantly associated with depressive symptoms in bivariate analyses were entered into multivariable model through stepwise (forward conditional) method. We also conducted stepwise backward conditional method, where we got exactly the same result as in forward conditional method. The probabilities for entry and removal of variable in each step were set as 0.05 and 0.1, respectively. Categorical variables were coded appropriately before entering them into the model.

## 3. Result

After excluding 11 participants who did not respond well, total participants were 379, with 50.1% females and 49.9% males. The mean age of the respondents was 54.8 ± 10.6 years with median duration of diabetes of 8.7 years (IQR = 10 years). The majority of the participants had age between 51 and 60 years (34.3%), were from nuclear family (85.8%) and urban area (84.7%), and belonged to Newar ethnic group (47%) and Hinduism (87.1%). Most of respondents were homemakers (29.8%) and from lower socioeconomic status (73.9%) (Table 1).

### 3.1. Reliability of the Instrument

The reliability of both instruments was assessed using the study dataset. Reliability coefficients (Cronbach alpha) were computed for each of the IPQ-R subscales and the BDI-II score. Among nine illness perception subscales, six had an adequate reliability coefficient (*α* levels > 0.70). Internal consistency of treatment control subscale was very low (*α* = 0.3). BDI-II scale, however, showed an excellent reliability with high Cronbach alpha (*α* = 0.869) (Table 2).

### 3.2. Presence of Depressive Symptoms according to Sociodemographic Characteristics

The prevalence of depressive symptoms among the respondents was 44.1% (95% CI: 39.1, 49.1). It was significantly high among females compared to their male counterparts (*p* < 0.001). Also, proportions of depressive symptoms were high among homemakers (65.4%), patients with lower socioeconomic status (70.9%), and age group 61–70 years (58.2%) (Table 3).

### 3.3. Illness Perception

The average score of personal control was comparatively higher than mean score of other subscales (Table 3). It is because the majority (75%) of participants agreed that there was a lot which they could do to control their symptoms. Most of the respondents (82%) also believed the course of diabetes depended on them. In contrast to that, cyclical timeline had the lowest average score among all subscales. A large proportion of respondents (43%) disagreed that the symptoms of diabetes changed a great deal from day to day in cyclical nature.

### 3.4. Depressive Symptoms and Illness Perception

Spearman correlation analysis found that identity (*r* = 0.525, *p* < 0.001), acute timeline (*r* = 0.115, *p* = 0.025) and cyclical timeline (*r* = 0.337, *p* < 0.001), consequences (*r* = 0.511, *p* < 0.001), emotional representation (*r* = 0.636, *p* < 0.001), and causes (*r* = 0.366, *p* < 0.01) components had a significant positive correlation with BDI-II scores (Table 4). Contrarily, treatment control (*r* = −0.250, *p* < 0.001) and illness coherence (*r* = −0.446, *p* < 0.001) had a negative relationship with depressive symptoms (Table 4). In a separate Spearman correlation analysis, duration of diabetes had no significant relationship with depression scores (*r* = 0.013, *p* = 0.802).

In multivariable analysis, all explanatory variables significant in bivariate analysis were entered by a stepwise (forward conditional) method into binary logistic regression model. Seven variables, emotional representation (*β* = 0.214, *p* < 0.001), identity (*β* = 0.196, *p* < 0.001), no formal education (*β* = 1.959, *p* = 0.01), age (*β* = 0.036, *p* = 0.016), illness coherence (*β* = −0.109, *p* = 0.007), mode of treatment (*β* = 0.9, *p* = 0.047), and consequences (*β* = 0.093, *p* = 0.049), were finalized as significant predictors of depressive symptoms (Table 5). Multivariable analysis showed that one-year increase in age shifted odds of having depressive symptoms by 1.037. People without formal education had seven times higher chance of suffering from depressive symptoms than people with Master's degree. In the same way, treatment with insulin had higher likelihood of getting depressive symptoms than the people taking oral medication. People using insulin for treatment of their diabetes had tendency to suffer from depressive symptoms 2.3 times higher than people on medication. Likewise, illness perception related to identity, consequences, and emotional representation about diabetes had positive relationship with depressive symptoms. One-unit increase in identity, consequences, and emotional representation heightened the likelihood of getting depressive symptoms among diabetes patients by 21%, 9.8%, and 23.9%, respectively. On the other hand, the same amount increase in illness coherence was responsible for 10% decrease in depressive symptoms among diabetes patients.

## 4. Discussion

The current study found prevalence of depressive symptoms in diabetic patients to be 44.1%. This result was in line with the study conducted in Nepal in similar setting where the prevalence was seen to be 40.3% [14]. This rate was also comparable to prevalence of depression (36.6%) as reported in a meta-analysis of studies among persons with diabetes in clinical settings [32].

### 4.1. Depressive Symptoms in relation to Sociodemographic Factors

Our study showed that female respondents with lower socioeconomic status and lower educational background and homemakers were significantly associated with depressive symptoms. Various studies have reported that female, elderly people and single women are more susceptible to anxiety and depression [33–37]. A meta-analysis including 39 studies also demonstrated that the prevalence of depression in patients with diabetes was significantly high in women compared to men [10]. Similarly, a study conducted in hospital settings in India pointed out that depression was more prevalent in female as compared to males [38]. The high proportion of depressive symptoms in women might have been observed because of the presence of more females from low socioeconomic group, having less educational status, and spending most time as homemaker compared to men. Besides, low socioeconomic status, having less education, and spending most time as homemaker have their association with depression among diabetic patients independently [34, 36, 37]. This study clearly indicated that depressive symptoms did not have a significant disproportional distribution among different religions, family type, and ethnic groups. In the same way, this study also did not find a significant association of alcohol consumption and smoking with depressive symptoms, which is consistent with previous studies conducted in similar setting in Kathmandu, Nepal, and Tlemcen, Algeria [14, 39].

Similarly, presence of depressive symptoms was significantly high among the people who were using insulin for the treatment of diabetes compared to the participants using only medications. They might have perceived insulin treatment as the severity of condition as well as the burden to their daily activities. Katon et al. and Al-Amer et al. concluded that taking insulin for controlling diabetes had significant association with depression [34, 40]. Our study also could not show a significant relationship between depressive symptoms and duration of diabetes. This was consistent with other studies that reported no significant relationship of the duration of diabetes with depression [40–42].

### 4.2. Depressive Symptoms and Illness Perception

Our study demonstrated that illness perception of diabetic respondents was related to depressive symptoms they had. All the components of illness perception except personal control had significant relationship with depressive symptoms. This suggested participants with high depressive symptoms perceived diabetes as a condition having more symptoms. They also believed that their illness was more severe and cyclical in nature, had less effective treatment control, possessed more serious consequences, was less understandable, and consisted of many causal factors. In comparison to respondents having no depressive symptoms, they also expressed high emotional response. These findings were consistent with another study conducted among patients having end stage of renal disease that reported a significant corelation of identity, cyclical nature, consequences, personal control, treatment control, illness coherence, emotional response, and causes with depression [16]. Also, in researches conducted in rheumatoid arthritis and cardiac patients, perceiving serious illness consequences had negatively been correlated with depression [22, 43]. Our study could not substantiate the same study that reported personal control had a significant relation with depressive symptoms [22]. In our study, we found the majority of the participants believed they could control their symptoms. Irrespective of presence of strong confidence over the personal control of diabetes among the participants, the reason behind not having its significant relationship with depressive symptoms remained unclear.

This study also identified the illness identity, consequences, emotional representations, and coherence as the significant predictors of depressive symptoms. Patients who perceived more symptoms (identity), more consequences, high emotional response, and less understanding (coherence) of diabetes were likely to have more depressive symptoms than others. Findings were suggestive of the fact that patients were more likely to get depressed when they knew less about their condition. Similarly, patients with high depressive symptoms were likely to experience many symptoms of diabetes that were, in fact, not related to it. According to Leventhal's model of self-regulation, patients make their own lay views about disease after experiencing the symptoms and that cognition shapes their coping behaviors [17, 18, 44]. Therefore, familiarity with the symptoms and complications of any disease can make the patient more confident to deal with it [45], whereas poor perception leads to maladaptive outcomes of psychological distress [44, 46].

Overall, the current study identified a relationship between illness perception and depressive symptoms among diabetic patients. This study is the first to establish their relationship among persons with diabetes in Nepal. It has major implication for the development of patient centered intervention in prevention and management of depressive symptoms among Nepalese diabetic patients. Addressing illness perception early on persons with diabetes can provide the opportunity to improve their beliefs and can achieve significant positive influences on disease outcomes [45, 47]. However, it was only an analytical cross- sectional study. This negates the ability to decide the causality. It does not uncover the relationship of illness perception and glycemic control in diabetic patients. As many studies reported the significant association of glycemic control with depression [48], understanding the tripartite relationship of illness perception, glycemic control, and depression among diabetic patients could be an interesting area of study in future. Similarly, the poor internal consistency of treatment control and personal control IPQ-R subscales is also concerning limitation of the study. But inadequate reliability coefficient of aforementioned two subscales seems to be a generalized problem. Several studies reported the same [49–51]. Lastly, samples were taken from clinical settings and could not represent community as a whole; a community based study is warranted for better representation.

## 5. Conclusion

This study underlines the importance of understanding of illness perception for preventing depressive symptoms that paves the way for effective treatment and control among type 2 diabetics. Providing information about the cause, pathology, complication, and overall treatment of disease can help to form a decent illness perception. Hospital and treatment centers should create a favorable environment where patients can have access to authentic and appropriate information and check the viability of their illness perceptions. This may help patients to follow active coping behaviors, ultimately preventing depressive symptoms and also helping in the treatment and control of diabetes.

## Figures and Tables

**Table 1:** Sociodemographic characteristics of respondents by gender.

Characteristics	Gender		Total (%)	*p* value
	Male (%)	Female (%)		
Age group (in years)				
20–30	3 (0.8)	2 (0.5)	5 (1.3)	0.15
31–40	15 (4)	27 (7.1)	42 (11.1)	
41–50	30 (7.9)	50 (13.2)	80 (21.1)	
51–60	74 (19.5)	56 (14.8)	130 (34.3)	
61–70	67 (17.7)	55 (14.5)	122 (32.2)	
Family type				
Nuclear	164 (43.3)	161 (42.5)	325 (85.8)	0.571
Joint	25 (6.6)	29 (7.7)	54 (14.2)	
Place of residence				
Urban	152 (40.1)	169 (44.6)	321 (84.7)	0.021
Rural	37 (9.8)	21 (5.5)	58 (15.3)	
Ethnicity				
Chhetri	29 (7.7)	32 (8.4)	61 (16.1)	0.005^*∗*^
Brahman	58 (15.3)	29 (7.7)	87 (23)	
Newar	83 (21.9)	95 (25.1)	178 (47)	
Janajati	13 (3.4)	25 (6.6)	38 (10)	
Others	6 (1.6)	9 (2.4)	15 (4)	
Religion				
Hindu	175 (46.2%)	155 (40.9)	330 (87.1)	0.001^*∗*^
Others	14 (3.7)	35 (9.2)	49 (12.9)	
Occupation				
Job	59 (15.6)	21 (5.5)	80 (21.1)	0.00^*∗∗∗*^
Business	48 (12.7)	21 (5.5)	69 (18.2)	
Farming	26 (6.9)	11 (2.9)	37 (9.8)	
Homemaker	3 (0.8)	110 (29)	113 (29.8)	
Jobless	20 (5.3)	10 (2.6)	30 (7.9)	
Others	33 (8.7)	17 (4.5)	50 (13.2)	
Education level				
No formal education	9 (2.4)	75 (19.8)	84 (22.2)	0.00^*∗∗∗*^
Less than primary	3 (0.8)	4 (1.1)	7 (1.8)	
Primary	27 (7.1)	43 (11.3)	70 (18.5)	
Secondary	49 (12.9)	32 (8.4)	81 (21.4)	
Higher secondary	30 (7.9)	16 (4.2)	46 (12.1)	
Bachelor's degree	42 (11.1)	14 (3.7)	56 (14.8)	
Master's degree	29 (7.7)	6 (1.6)	35 (9.2)	
Socioeconomic status				
Lower	12 (3.2)	98 (25.9)	110 (29)	0.00^*∗∗∗*^
Upper lower	97 (25.6)	73 (19.3)	170 (44.9)	
Lower middle	44 (11.6)	8 (2.1)	52 (13.7)	
Upper middle	36 (9.5)	11 (2.9)	47 (12.4)	
Upper	—	—		
Marital status				
Married	186 (49.1)	155 (40.9)	341 (90)	0.00^*∗∗∗*^
Others	3 (0.8)	35 (9.2)	38 (10)	

^*∗*^ *p* < 0.05, ^*∗∗*^ *p* < 0.01, and ^*∗∗∗*^ *p* < 0.001.

**Table 2:** Reliability test (Cronbach alpha) for illness perception and BDI-II scale.

Scales	Mean	SD	Cronbach alpha value
Identity	6.6	3.7	0.738
Timeline (acute and chronic)	21.5	3.8	0.83
Consequences	17.7	3.8	0.644
Personal control	22.7	2.4	0.5
Treatment control	16.9	2.2	0.3
Illness coherence	16.1	3.9	0.828
Timeline (cyclical)	11.7	3.4	0.785
Emotional representation	17.9	4.9	0.839
Cause	49.7	7.5	0.726
Depression (BDI-II) scale	12.9	8.1	0.869

**Table 3:** Distribution of depressive symptoms by sociodemographic characteristics and behavior factors.

Characteristics	Depressive symptoms		Total	*p* value
	No (%)	Yes (%)		
Age group				
20–30	2 (0.5)	3 (0.8)	5 (1.3)	0.001^*∗∗*^
31–40	27 (7.1)	15 (4)	42 (11.1)	
41–50	45 (11.9)	35 (9.2)	80 (21.1)	
51–60	87 (23)	43 (11.3)	130 (34.3)	
61–70	51 (13.5)	71 (18.7)	122 (32.2)	
Sex				
Male	131 (34.6)	58 (15.3)	189 (49.9)	0.000^*∗∗∗*^
Female	81 (21.4)	109 (28.8)	190 (50.1)	
Family type				
Nuclear	187 (49.3)	138 (36.4)	325 (85.8)	0.123
Joint	25 (6.6)	29 (7.7)	54 (14.2)	
Place of residence				
Urban	181 (47.8)	140 (36.9)	321 (84.7)	0.678
Rural	31 (8.2)	27 (7.1)	58 (15.3)	
Ethnicity				
Chhetri	37 (9.8)	24 (6.3)	61 (16.1)	0.347
Brahman	47 (12.4)	40 (10.6)	87 (23)	
Newar	102 (26.9)	76 (20.1)	178 (47)	
Janajati	16 (4.2)	22 (5.8)	38 (10)	
Others	10 (2.6)	5 (1.3)	15 (4)	
Religion				
Hindu	185 (48.8)	145 (38.3)	330 (87.1)	0.9
Others	27 (7.1)	22 (5.8)	49 (12.9)	
Occupation				
Job	58 (15.3)	22 (5.8)	80 (21.1)	0.000^*∗∗∗*^
Business	49 (12.9)	20 (5.3)	69 (18.2)	
Farming	19 (5)	18 (4.7)	37 (9.8)	
Homemaker	39 (10.3)	74 (19.5)	113 (29.8)	
Jobless	16 (4.2)	14 (3.7)	30 (7.9)	
Others	31 (8.2)	19 (5)	50 (13.2)	
Education level				
No formal education	18 (4.7)	66 (17.4)	84 (22.2)	0.000^*∗∗∗*^
Less than primary	4 (1.1)	3 (0.8)	7 (1.8)	
Primary	32 (8.4)	38 (10)	70 (18.5)	
Secondary	55 (14.5)	26 (6.9)	81 (21.4)	
Higher secondary	32 (8.4)	14 (3.7)	46 (12.1)	
Bachelor's degree	45 (11.9)	11 (2.9)	56 (14.8)	
Master's degree	26 (6.9)	9 (2.4)	35 (9.2)	
Socioeconomic status				
Lower	32 (8.2)	78 (20.6)	110 (29)	0.000^*∗∗∗*^
Upper lower	98 (25.9)	72 (19)	170 (44.9)	
Lower middle	45 (11.9)	7 (1.8)	52 (13.7)	
Upper middle	37 (9.8)	10 (2.6)	47 (12.4)	
Marital status				
Married	198 (52.2)	143 (37.7)	341 (90)	0.012^*∗*^
Others	14 (3.7)	24 (6.3)	38 (10)	
Alcohol consumption				
Yes	31 (8.2)	14 (3.7)	45 (11.9)	0.062
No	181 (47.8)	153 (40.4)	334 (88.1)	
Smoking				
Yes	25 (6.6)	11 (2.9)	36 (9.5)	0.86
No	187 (49.3)	156 (41.2)	343 (90.5)	
Mode of treatment				
Insulin	21 (5.5)	37 (9.8)	58 (15.3)	0.001^*∗∗*^
Oral medication	191 (50.4)	130 (34.3)	321 (84.7)	

^*∗*^ *p* < 0.05, ^*∗∗*^ *p* < 0.01, and ^*∗∗∗*^ *p* < 0.001.

**Table 4:** Relationship between depressive symptoms and illness perception (Spearman correlation).

	Depression	Identity	Timeline (acute)	Timeline (cyclic)	Consequences	Personal control	Treatment control	Illness coherence	Emotional representation	Cause
Depression (BDI-II) scale	1									
Identity	0.525^*∗∗*^	1								
Timeline (acute)	0.115^*∗*^	0.106^*∗*^	1							
Timeline (cyclic)	0.337^*∗∗*^	0.328^*∗∗*^	0.35	1						
Consequences	0.511^*∗∗*^	0.410^*∗∗*^	0.170^*∗∗*^	0.437^*∗∗*^	1					
Personal control	−0.099	−0.087	−0.077	−0.127^*∗*^	−0.108^*∗*^	1				
Treatment control	−0.250^*∗∗*^	−0.231^*∗∗*^	−0.435^*∗∗*^	−0.074	−0.247^*∗∗*^	0.225^*∗∗*^	1			
Illness coherence	−0.446^*∗∗*^	−0.305^*∗∗*^	−0.018	−0.515^*∗∗*^	−0.356^*∗∗*^	0.193^*∗∗*^	0.166^*∗∗*^	1		
Emotional representation	0.636^*∗∗*^	0.412^*∗∗*^	0.018	0.477^*∗∗*^	0.518^*∗∗*^	−101^*∗*^	−0.156^*∗∗*^	−0.468^*∗∗*^	1	
Cause	0.366^*∗∗*^	0.390^*∗∗*^	0.006	0.162^*∗∗*^	0.395^*∗∗*^	−0.081	−0.069	−0.252^*∗∗*^	0.342^*∗∗*^	1

^*∗*^ *p* < 0.05, ^*∗∗*^ *p* < 0.01, and ^*∗∗∗*^ *p* < 0.001.

**Table 5:** Multivariable analysis of depressive symptoms (binomial logistic regression, stepwise method).

Variables	Category	*B*	*p* value	Exp(*B*)
Constant		−8.223	0.000^*∗∗∗*^	0.000

Age in years		0.036	0.016	1.036

Education level	Master's degree and higher	Reference	0.003^*∗∗*^	
	No formal education	1.959	0.001^*∗∗*^	7.095
	Less than primary	0.131	0.902	1.140
	Primary	0.651	0.257	1.917
	Secondary	0.052	0.928	1.053
	Higher secondary	0.578	0.366	1.782
	Bachelor's degree	0.183	0.783	1.201

Mode of treatment	Medicine	Reference		
	Insulin	0.900	0.047^*∗*^	2.307

Identity		0.196	0.000^*∗∗∗*^	1.216

Consequences		0.093	0.049^*∗*^	1.098

Illness coherence		−0.109	0.007^*∗∗*^	0.896

Emotional representation		0.214	0.000^*∗∗∗*^	1.239

^*∗*^ *p* < 0.05, ^*∗∗*^ *p* < 0.01, and ^*∗∗∗*^ *p* < 0.001.
